# Electronic Structure and Room Temperature Ferromagnetism in Gd‐doped Cerium Oxide Nanoparticles for Hydrogen Generation via Photocatalytic Water Splitting

**DOI:** 10.1002/gch2.201800090

**Published:** 2019-01-07

**Authors:** Swati Soni, Neelu Chouhan, Rajesh Kumar Meena, Sudhish Kumar, Bhavna Dalela, Monu Mishra, Rajendra Singh Meena, Govind Gupta, Shalendra Kumar, Parvez Ahmad Alvi, Saurabh Dalela

**Affiliations:** ^1^ Department of Pure and Applied Physics University of Kota Kota 324005 Rajasthan India; ^2^ Department of Pure and Applied Chemistry University of Kota Kota 324005 Rajasthan India; ^3^ Department of Physics Mohan Lal Sukhadia University Udaipur Rajasthan India; ^4^ Department of Physics Govt. Khetan Polytechnic College Jhalana Dungri Jaipur Rajasthan India; ^5^ CSIR‐National Physical Laboratory Dr. K. S. Krishnan Road New Delhi 110012 India; ^6^ Electronic Materials and Nanomagnetism Lab Department of Applied Physics Amity School of Applied Sciences Amity University Haryana Gurgaon 122413 Haryana India; ^7^ Department of Physics Banasthali University Newai 304022 Rajasthan India

**Keywords:** hydrogen generation, SERS, water splitting, XPS

## Abstract

Enhanced visible light photocatalytic activity of Gd‐doped CeO_2_ nanoparticles (NPs) is experimentally demonstrated, whereas there are very few reports on this mechanism with rare earth doping. All‐pure and Gd‐doped CeO_2_ NPs are synthesized using a coprecipitation method and characterized using X‐ray diffraction (XRD), absorption spectroscopy, surface‐enhanced Raman Spectroscopy (SERS), X‐ray photoelectron spectroscopy (XPS), and superconducting quantum interference device (SQUID). The effect of Gd‐doping on properties of CeO_2_ is discussed along with defects and oxygen vacancies generation. The XRD confirms the incorporation of Gd^3+^ at the Ce^3+^/Ce^4+^ site by keeping the crystal structure same. The average particle size from transmission electron microscopy (TEM) images is in the range of 5–7 nm. The XPS spectra of Ce 3d, O 1s, and Gd 4d exhibits the formation of oxygen vacancies to maintain the charge neutrality when Ce^4+^ changes to Ce^3+^. The gradual increase in hydrogen production is observed with increasing Gd concentration. The observed results are in good correlation with the characterization results and a mechanism of water splitting is proposed on the basis of analyses. The absorption spectra reveal optical band gap (2.5–2.7 eV) of samples, showing band gap narrowing leads to desired optical absorbance and photoactivity of NPs.

## 1 Introduction

Rare earth (RE) oxide‐CeO_2_ has attracted great interest of research due to their unique properties, including high oxygen storage capacity and ability to uptake and release oxygen (O^2−^) ions via conversion of oxidation state of cerium ion from Ce^4+^ to Ce^3+^, due to formation of defect space such as oxygen vacancies in the lattice of CeO_2_.^1^ On account of this uniqueness, CeO_2_ has been widely used as three‐way catalysts for eliminating vehicle exhaust gases,^2^ UV blocker materials in sunscreens, UV‐shielding used in cotton fabrics,^3^ functionalize silk fiber for antibacterial activity,^4^ oxygen sensors,^5^ and oxygen ion conductors in solid oxide fuel cells (SOFCs).^6^ Generally, it has been reported that type of dopant strongly influenced the electrical properties of ceria and high conductivity at low temperature is an essential requirement for SOFCs, therefore, rare earth (RE)‐doped cerium oxide, Ce_1−*x*_RE_*x*_O_2−δ_ (RE = Sm, Gd, Dy, Er, Lu) are preferable dopants used as electrolytes for intermediate‐temperature SOFCs.^7^, ^8^ Alike TiO_2,_ bulk cerium dioxide (CeO_2_) is a wide band gap (3.2 eV) cubic fluorite semiconductor,^9^ which possesses the interesting properties such as a high dielectric constant (ε = 26), good transparency in the visible range, nontoxic, and capacity to exhibit the high photocatalytic activity under UV light irradiation. Therefore, CeO_2_ seems to be a promising inorganic material that can be used for the UV filtration in sunscreen/cosmetic products and as a potential material for UV filtration.^10^ Therefore, to enhance the workability of this compound in visible light some kind of structural engineering and doping of heavy metal might be done for reducing the band gap of CeO_2_.

Among all RE‐doped CeO_2_, Sm and Gd‐stabilized ceria has been extensively studied for utilization as electrolyte and anode material.^11^, ^12^ It has been reported that addition of Sm^3+^ and Gd^3+^ cations in CeO_2_ system produced highest conductivity with least distortion of parent lattice, which is attributed to the smallest association enthalpy between the dopant cation and the oxygen vacancies in the CeO_2_ lattice.^13^, ^14^, ^15^ Besides of dopant type, theoretical and experimental observations have also suggested that the ionic conduction can be altered by the concentration of dopant.^16^, ^17^, ^18^, ^19^ Moreover, it is well known that material properties changes when particle size reduces to nanoscale, as reported by Kosacki et al.^20^ in their nanocrystalline CeO_2_ thin film, electrical conductivity has found to be enhanced due to reduced enthalpy of oxygen vacancy formation. Li et al.^21^ have reported increase in catalytic activity as well as in optical and magnetic properties of porous Gd^3+^‐doped CeO_2_ (10 at% Gd) nanostructure due to Gd^3+^ ions or formation of oxygen vacancies. In most of these studies, the local ordering of oxygen vacancies on grain boundaries in heavily RE‐doped CeO_2_ samples has been reported.^22^, ^23^, ^24^ Chen et al.^25^ have reported dopant‐induced structural differences and defects in Sm‐doped CeO_2_ nanoparticles (NPs) with doping concentration 3%, 5%, 7%, 9%, and 11%. On the basis of their results based on X‐ray absorption spectroscopy, extended X‐ray absorption fine structure, Raman, and scanning transmission electron microscope‐electron energy loss spectroscopy measurements they have discussed that below 7% and above 7% distribution of defects strongly depend on the concentration of Sm^3+^ ions in CeO_2_ NPs. Since, there is a lack of literature available on the evidence of the distribution of defect study with small doping concentration of RE‐doped CeO_2_ NPs.

Metal oxide photocatalyst have attracted increasing attention due to their potential applications in the environmental protection and energy utilization, such as water splitting for hydrogen production. In the Zn‐, Mg‐, and Ca‐doped CeO_2_ materials the impurities tend to shift the band position and can tune the band gap because of their effects on electronic transition.^26^ Gd‐doped CeO_2_ is used for thermolysis of water that can produce 101.6 H_2_ (µmol g^−1^) hydrogen.^27^ It has been demonstrated experimentally that the rare earth dopants and oxygen vacancies greatly influenced the photocatalytic properties of CeO_2_; however, the effect of interaction between the rare earth dopant and oxygen vacancy defects on enhanced visible light photocatalytic activity of CeO_2_ is still not investigated so far.

Hence in this study, we systematically explore to develop the correlation between the rare earth dopants, its concentration and oxygen vacancy defects to enhance the photocatalytic activity of doped CeO_2_. To undertake this study we have investigated the structural properties, dopant distribution, and their association with oxygen vacancies in Gd‐doped CeO_2_ NPs. The CeO_2_ NPs have been doped with different concentrations of Gd^3+^ ions (2%, 4%, 6%, 8%, and 10%) to discuss the presence of defect induced oxygen vacancies (either intrinsic or extrinsic) and their association with doped cation with surface‐enhanced Raman spectroscopy (SERS) measurements. Transmission electron microscopy (TEM) has been used to observe changes in the surface morphology and particle size with increased fluencies of Gd^3+^ ions in CeO_2_ lattice. The optical absorption spectra have been measured using ultraviolet–visible–near infrared (UV–Vis–NIR) spectrometer to find out the band gap energy. Finally, we have attempted to investigate how doping concentration affect the oxygen vacancies and cation (Ce^3+^) defects, present in pure and Gd‐doped CeO_2_ NPs. These results offer a physical understanding for the available experimental results to explain the enhanced photocatalytic activities of Gd‐doped CeO_2_ NPs, which can be useful for designing and understanding the novel doped CeO_2_ photocatalyst.

## 2 Result and Discussion

### 2.1 X‐Ray Diffraction (XRD) Analysis

**Figure 1:**
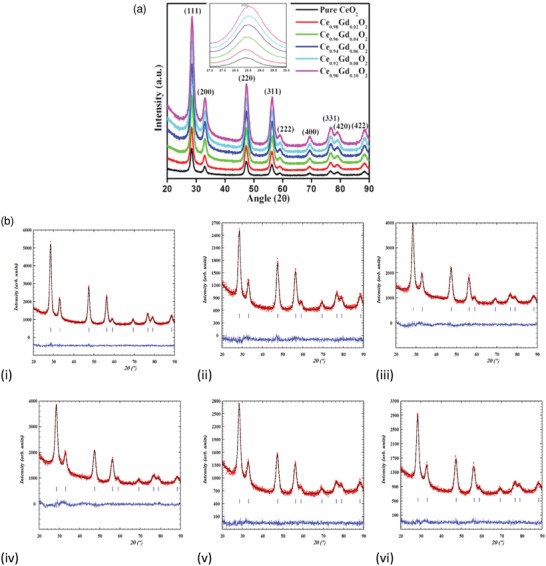
a) XRD pattern of pure CeO_2_ and Ce_1−*x*_Gd_*x*_O_2_ (for *x* = 0.02, 0.04, 0.06, 0.08, and 0.10) samples. b) Refined and fitted X‐ray diffraction patterns of Ce_1−_ *_x_*Gd*_x_*O_2_ at 300 K (i) *x* = 0.00 (ii) *x* = 0.02, (iii) *x* = 0.04, (iv) *x* = 0.06, (v) *x* = 0.08, and (vi) *x* = 0.10. Observed (calculated) profiles are shown by dotted (solid) lines. The short vertical marks represent Bragg reflections. The lower curve is the difference plot.

X‐ray diffraction measurements have been made on Ce_1−*x*_Gd_*x*_O_2_ nanoparticles for *x* = 0.0, 0.02, 0.04, 0.06, 0.08, and 0.10 at room temperature are shown in **Figure** **1**a. Nanocrystalline Gd‐doped CeO_2_ samples exhibit fundamental Bragg reflections corresponding to the fluorite type face centered cubic structure in the space group of *Fm‐3m*, in which Ce and Gd atoms are located at 4a position, surrounded by eight O (located at 8b) positions.^28^ Absence of any secondary phase corresponding to Gd_2_O_3_ or other impurity peaks indicates well incorporation of Gd^3+^ ions on CeO_2_ lattice site, which confirms the single phase formation of all the Ce_1−*x*_Gd_*x*_O_2_ nanoparticles. The intensity of XRD diffraction peaks is found to vary with incorporation of Gd^3+^ ions in CeO_2_ NPs (as shown in Figure 1a).

The rising intensity signifies an improvement in the crystalline nature while falling intensity signifies low crystallinity of Ce_1−*x*_Gd_*x*_O_2_ samples.^29^ Moreover, with fluency of Gd^3+^ ions, no peak shifting is observed for *x* = 0.02 doping concentration, whereas with increasing concentration (*x* ≤ 0.06) diffraction peak (111) is shifted toward higher angle side (as shown inset of Figure 1a). For higher doping concentrations (*x* = 0.08 and 0.10) peak is again shifted toward lower angle side. This shifting of (111) peak toward lower and higher angle side is attributed to the lattice expansion and reduction, respectively, which is induced by incorporation of Gd^3+^ ions in CeO_2_ NPs.^30^ Furthermore, it can be seen in Figure 1a, diffraction peaks become broader after doping and broadness of peaks are also observed to change with fluency of Gd^3+^ ions in CeO_2_ NPs, indicating that crystal size and crystallinity of the samples are affected with the fluencies of Gd^3+^ ions. The average nanocrystalline particle size (*D*) of Gd‐doped CeO_2_ samples has been calculated with XRD diffraction spectra using the Debye–Scherrer's formula^31^

(1) $$D\;=\frac{k\;\lambda }{\beta \;\cos\theta }$$

where, all the parameters are as per the details given in ref. 15. The lattice parameters of all the samples corresponding to (111) diffraction peak have been calculated by the following formula^32^

(2) $$a\;=\;d\;{\left(h^{2}+k^{2}+l^{2}\right)}^{1/2}$$

where “*a*” refers to the lattice parameter, *d* is the crystalline lattice spacing, and *h*, *k*, *l*, are the miller indices of crystal. The calculated value of lattice parameter with fluency of Gd^3+^ ions in CeO_2_ NPs is tabulated in **Table** **1**.

**Table 1:** Calculated values of lattice parameter (*a*), lattice spacing (*d*) for (111) plane, average crystalline size (*D*) measured from TEM, XRD line broadening, and Raman line broadening, dislocation density (δ), lattice strain (ε), absorbance wavelength (λ), optical band gap energy (*E*_g_), and refractive index (*n*) are summarized in this table

Sample	Parameters									
	*a* [Å]	*d* [nm]	*D* [nm]			$\delta =\frac{1}{D^{2}}$ [nm^−2^] 33	ε = $\frac{\beta \;\cos\theta }{4}$ × 10^−2^ 34, 35	λ [nm]	*E*_g_ [eV]	*n*
			TEM	XRD	Raman spectra					
Pure CeO_2_	5.436	0.318	5.55	9.21	10.9	0.0324	3.88	367	2.60	2.51
Ce_0.98_Gd_0.02_O_2_	5.433	0.307	5.27	6.95	7.52	0.0360	8.60	363	2.66	2.49
Ce_0.96_Gd_0.04_O_2_	5.392	0.318	6.44	7.10	6.45	0.0241	8.89	359	2.71	2.48
Ce_0.94_Gd_0.06_O_2_	5.394	0.316	7.47	6.51	6.07	0.0179	7.82	365	2.67	2.49
Ce_0.92_Gd_0.08_O_2_	5.393	0.324	5.55	6.22	5.66	0.0324	14.18	368	2.64	2.50
Ce_0.90_Gd_0.10_O_2_	5.398	0.312	6.34	6.26	5.06	0.0248	9.11	371	2.52	2.54

The variation in the calculated values of lattice parameter can be directly related to the ionic radii of the dopant ion. Since, the larger ionic radii Gd^3+^ (0.1053 nm) ions are substituted for the smaller ionic radii Ce^4+^ (0.097 nm) ions and created the larger radii Ce^3+^ ions (0.114 nm).^36^ Furthermore, for maintaining charge neutrality in CeO_2_ lattice, Gd^3+^ and Ce^3+^ ions are collectively creating oxygen vacancies in the CeO_2_ lattice, which causes further lattice expansion.^30^

Rietveld profile refinements (shown in Figure 1b(i–vi)) of all the samples are carried out and the results are listed in Table 1. The XRD patterns indicate that Gd‐doping in CeO_2_ does not affect the cubic fluorite structure of the CeO_2_, as no additional diffraction peaks related to possible impurity phases of Gd and oxides of Gd are observed in these Gd‐doped CeO_2_ samples. It is further confirmed the formation of a single phase of Ce_1‐_ *_x_*Gd*_x_*O_2_. The refinement results clearly indicate that Gd ions are well incorporated in the CeO_2_ matrix and Gd‐doping in CeO_2_ leads to small enhancement in the unit‐cell volume.

It is clear from Table 1, the dislocation density is found to increase for *x* = 0.02 doping concentration but decreases for *x* = 0.04 and 0.06, which is again increased for *x* = 0.08 and decreased for *x* = 0.10 doping concentrations of Gd^+3^ cation. This variation in dislocation density is related to the promotion and reduction of disorder in the crystal structure.^29^

Looking to Table 1, the values of strain indicates tensile strain for Gd‐doped CeO_2_ NPs. Due to incorporation of Gd^3+^ (0.1053 nm) cations in CeO_2_ NPs, the maximum value of strain is for *x* = 0.08 doping concentration. Some theoretical investigation revealed that tensile strain promotes the formation of oxygen vacancies rather than compressive strain.^37^ Therefore, in Gd‐doped CeO_2_ samples, increased value of tensile strain can be directly related to the endorsement of oxygen vacancies in doped CeO_2_ samples, which may be associated to the bonding length and the strength between the surface O and Ce atoms.^38^ Since, for tensile strain, the bandwidth of the O 2p orbital decreases and overlapping between O 2p and Ce 5d as well as 4f orbital also decreases, which leads a weaker Ce—O bond and responsible for the formation of oxygen vacancies in doped CeO_2_ system.^37^ The crystallinity, morphology, and particle size of the Gd‐doped CeO_2_ samples are discussed in the next segment by TEM, high‐resolution transmission electron microscopy (HRTEM), and selected area (electron) diffraction (SEAD) images.

### 2.2 Surface Morphology

**Figure 2:**
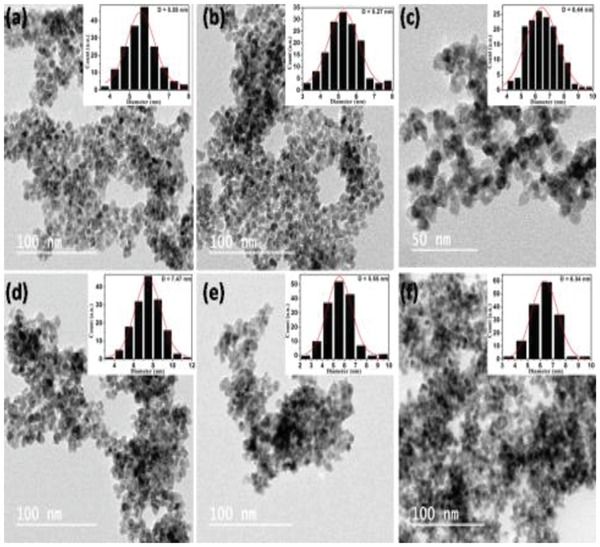
TEM image for Gd‐doped CeO_2_. a) Pure CeO_2_, b) 2% Gd‐doped CeO_2_, c) 4% Gd‐doped CeO_2_, d) 6% Gd‐doped CeO_2_, e) 8% Gd‐doped CeO_2_, f) 10% Gd‐doped CeO_2_ and inset histogram graphs show the particle size of the corresponding sample.

**Figure 3:**
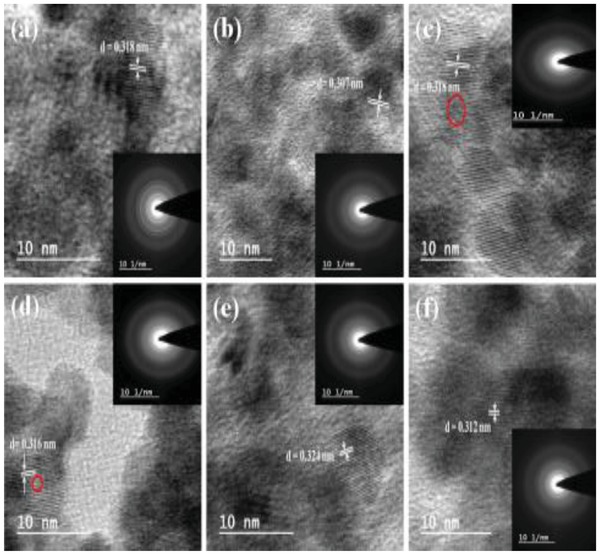
HRTEM images of Gd‐doped CeO_2_ with *d*‐spacing for (111) plane. a) Pure CeO_2_, b) 2% Gd‐doped CeO_2_, c) 4% Gd‐doped CeO_2_, d) 6% Gd‐doped CeO_2_, e) 8% Gd‐doped CeO_2_, f) 10% Gd‐doped CeO_2_ and inset graphs show the SAED pattern of corresponding sample.

The average crystallite particle size of all the samples is confirmed by electron microscopy investigations. TEM measurement is used to manifest the information about the shape, size, and the presence of any secondary phase in pure and Gd‐doped CeO_2_ NPs. The particle size and morphology of pure and Gd‐doped CeO_2_ NPs are analyzed by TEM as shown in Figure 3. From TEM analysis, it is observed that the particles are crystallized nanoparticles and agglomerated with spherical morphology. The average particle size calculated from TEM images are ranging from 5 to 7 nm for pure and Gd‐doped CeO_2_ NPs, which are in good agreement with the results obtained from Debye–Scherrer formula (listed in Table 1). It can be observed from TEM images that crystal growth is promoted with doping concentration of Gd‐ions. However, the morphology of all samples is not changing but the agglomeration of particles is increased with the doping concentration of Gd‐ions (as shown in **Figure** **2**).

The particle‐size distribution histogram (shown in the inset of Figure 2) shows that the distribution is quite narrow in the size range of 5–7 nm for Gd‐doped CeO_2_ NPs. This agglomeration of particles with smaller particle‐size (<7 nm) indicates that the obtained particles are nanocrystalline. Furthermore, HRTEM and SAED analysis are also used to decipher the information about the nanocrystallinity and impurity phases, if any present in Gd‐doped CeO_2_ NPs. HRTEM images (shown in **Figure** **3**) indicate that the lattice fringes are well developed and randomly oriented with respect to each other. Most of the lattice fringes of Gd‐doped CeO_2_ samples are about at a distance of 0.31 nm (values are tabulated in Table 1) that corresponding to the (111) lattice plane of the fluorite like cubic structure.

As shown in Table 1, no significant change is observed in the interplanar distance (*d*) for Gd‐doped CeO_2_ samples but for 8% Gd‐doped CeO_2_ sample, the interplanar distance (*d* = 0.32 nm for (111) plane) is slightly increased, which again promotes the crystal growth and indicates the low crystallinity. Some defects, such as dislocations (shown in Figure 3c,d, marked with a red ring) are also observed in the HRTEM image of 4% and 6% Gd‐doped CeO_2_ samples. Moreover, SAED patterns are also taken (shown in the insets of Figure 3) for Gd‐doped CeO_2_ samples. SAED pattern exhibits four broad rings, which could be attributed to (111), (200), (220), and (311) planes. These rings indicate that the particles are crystallized and diffraction rings are very well matched with the XRD measurement results. The fluency of Gd^3+^ ions in CeO_2_ NPs also affect the optical band gap energy, which is further discussed in the next segment by UV–Vis–NIR spectroscopy.

### 2.3 Optical Absorption

**Figure 4:**
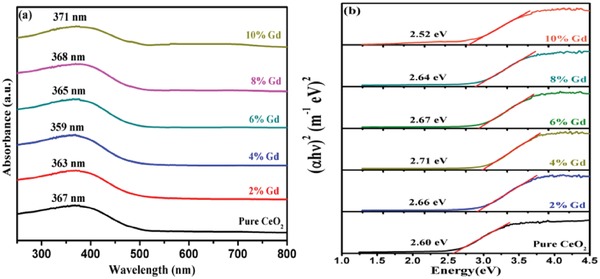
a) Room temperature optical absorbance spectra of pure CeO_2_ and Ce_1−*x*_Gd_*x*_O_2_ (*x* = 0.02, 0.04, 0.06, 0.08, and 0.10) samples taken in the UV–vis range. b) Tauc's plot of (*αhυ*)^2^ versus energy (eV) for the pure CeO_2_ and Gd‐doped CeO_2_ nanocrystalline samples.

**Figure** **4**a shows the UV–Vis–NIR absorption spectra of Ce_1−*x*_Gd_*x*_O_2_ samples (*x* = 0.00, 0.02, 0.04, 0.06, 0.08, and 0.10). These samples exhibit a strong absorption below 400 nm with an absorption peak in UV‐range corresponding to the different doping concentration of Gd^3+^ ions in CeO_2_ NPs as tabulated in Table 1. These peaks are originated due to direct charge transfer transition from 2p valance band of O^2−^ to 4f conduction band of Ce^4+^ ions.^39^ It is well known that CeO_2_ have wide band gap semiconductor with a forbidden gap of 5.5 eV.^40^ The valance band consists of O 2p level with a width of 4 eV and conduction band consist of Ce 5d level. Ce 4f level is present in between these two states, just above the Fermi level, that lies about 3 eV higher than the valance band (O 2p).^41^ Hence there is direct recombination of the electrons in Ce^4+^ (4f) conduction band with the holes in the O^2−^ (2p) valance band. It can be seen from Figure 4a, the absorbance peak is obtained at 367 nm for pure CeO_2_ but after incorporation of Gd^3+^ ions peak is shifted toward lower wavelength (blue shift) up to optimal doping concentration *x* = 0.04, while for further fluencies *x* = 0.06 to 0.10 peak is shifted toward higher wavelength (red shift) side. It has been reported that when metal NPs are forming smaller size particles, the λ_max_ shifts toward shorter wavelength (blue shift) side, whereas, when the smaller particles aggregate to form bigger/larger size particles, the λ_max_ value shifts toward longer wavelength (red shift) side.^42^ This may indicate that smaller‐sized particles have been formed up to doping concentration (*x* = 0.04), while with increasing fluencies of Gd‐ions (for *x* = 0.06 to 0.10) in CeO_2_ NPs, these smaller sized particles are agglomerated (as shown in Figure 2). As Hu et al. reported that the agglomeration of nanoparticles occurs because nanoparticles have a tendency to decrease the exposed surface in order to lower the surface energy, which results decreases in particle size with strong agglomeration.^43^ Furthermore, blue shifting in the absorption spectra with the fluency of Gd^3+^ ions in CeO_2_ NPs can be due to change of Ce^4+^ to Ce^3+^ state, that increases the direct charge‐transfer transition gap between O 2p and Ce 4f bands and reduces the particle size.^21^, ^44^ In addition of that the average particle size obtained from TEM images for Gd‐doped CeO_2_ NPs is in the range of 5–6 nm at lower doping concentration (*x* = 0.02 and 0.04), which is smaller than the predictable Bohr exciton radius for CeO_2_ (7–8 nm).^21^, ^45^ Therefore, the quantum confinement effect may also be taken place that contributes to the blue shift of the absorption spectra with small fluencies of Gd‐ions in CeO_2_ NPs. Generally, quantum confinement effect results when the Bohr radius of an exciton approaches the grain or particle size, spatially confining the electron–hole pair. When this happens, the energy of the lowest excited state increases and the increased band gap produces a blue shift in the absorption spectra. Now at the higher fluencies (for *x* = 0.06 to 0.10) of Gd^3+^ ions, the contribution of blue shifting from Ce^4+^ to Ce^3+^ valance state change will become small. Therefore, red shifting is occurred in the absorption spectra of Gd‐doped CeO_2_ (for 6%, 8%, and 10%) samples. This red shifting may be the outcome of an interfacial polaron effect arising from electron–phonon coupling phenomenon.^46^, ^47^ Form all above absorption data, the band gap energy (*E*_g_) of pure CeO_2_ and Ce_1−*x*_Gd_*x*_O_2_ (*x* = 0.02, 0.04, 0.06, 0.08, and 0.10) NPs has been calculated using Tauc's equation

(3) $$\alpha h\nu =A{\left(h\nu -E_{g}\right)}^{n}$$

where all the parameters have their usual meaning. For direct transition *n* = 1/2 and for *n* = 2 for indirect transition.^48^ Figure 4b displays the measured values of (*αhν*)^2^ as a function of the incident photon energy (*hν*). Table 1 contains the calculated value of band gap energy (*E*_g_) of all samples. Pure CeO_2_ NPs shows band gap energy of 2.60 eV that is smaller than the band gap energy reported for bulk ceria, i.e., 3.15 eV.^49^ This decrease in band gap energy may be attributed due to increase in the concentration of Ce^3+^ states on grain boundaries. Moreover, the optical band gap energy is found to increase for low fluency of Gd^3+^ ions (for *x* = 0.02 and 0.04) in CeO_2_ NPs while it decreases subsequently with increasing fluencies of Gd^3+^ ions (for *x* = 0.06 to 0.10) in CeO_2_ NPs (as shown in Table 1). This blue shift in the band gap energy at lower doping concentration (for *x* = 0.02 and 0.04) of Gd^3+^ ions may be correlated with the decrease of Ce^3+^ concentration as well as oxygen vacancies during annealing process. This may eliminate some localized defect energy states within the band gap due to the corresponding decrease of vacancies content, which results increase in the band gap energy.^50^ Another reason for explaining the increase in band gap energy may be correlated with the Burstein–Moss (BM) shift^49^

(4) $$\Delta E_{g}^{\mathrm{BM}}=\frac{h^{2}}{2m_{\mathrm{vc}}^{*}}\;{\left(3\pi^{2}n_{e}\right)}^{2/3}$$

Here, $m_{\mathrm{vc}}^{*}$ is effective mass of electrons, *n*_e_ is the electron concentration, and *h* is the Plank constant. Now, according to BM effect, above the Mott critical density, the increased number of free electron concentration is leading to fill 4f level partially, which in turn blocks the lowest states and leads to band gap widening.^49^, ^51^ With incorporation of Gd^3+^ ions into CeO_2_ sample, the crystalline size is reduced (as shown in Table 1). Therefore, the charge carriers are more confined in the small sized particles, which in turn increasing the band gap energy at lower doping concentration of Gd‐doped CeO_2_ NPs. This implies that, both particle size and BM effect results in the increase in band gap energy. Besides that, the red shift in band gap energy with higher fluencies of Gd^3+^ ions (*x* = 0.06, 0.08, and 0.10) is caused with the existence of Ce^3+^ contents at the grain boundaries, which increases with decreasing particle size.^52^ The refractive index of Gd‐doped CeO_2_ NPs has been calculated by using the following formula^53^

(5) $$\frac{n^{2}-1}{n^{2}+2}=1-\sqrt{\frac{E_{g}}{20}}$$

**Figure 5:**
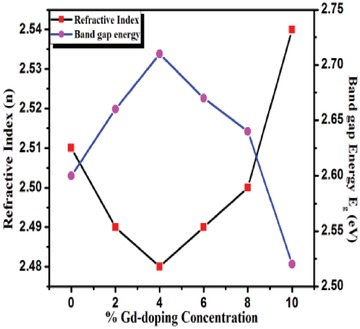
Variation of refractive index and bang gap energy with Gd‐doping concentration in nanocrystalline CeO_2_ samples.

The obtained values for the refractive index of pure and Gd‐doped CeO_2_ NPs are tabulated in Table 1. These values indicates that the refractive index is found to decrease with fluency of Gd ions up to optimal doping concentration *x* = 0.04, whereas it is increased for further fluencies (*x* = 0.06, 0.08, and 0.10) of Gd^3+^ ions in CeO_2_ NPs. The variation in the refractive index and band gap energy has been shown in **Figure** **5** with different concentration of Gd‐ions in CeO_2_ NPs. Therefore, absorption of UV light at low concentration of Gd‐ions (*x* = 0.02 and 0.04) in CeO_2_ NPs has been increased due to reduction of particle size as well as refractive index, whereas, due to increasing doping concentration the transparency and UV protection qualities are decreased.^54^

### 2.4 Raman Spectra

Surface enhanced Raman spectroscopy is a powerful vibrational technique, which allows for highly sensitivity structural detection of low concentration analyses through the amplification of electromagnetic fields generated by the excitation of localized surface plasmons.^55^ SERS provides the same information as traditional Raman spectroscopy does, but with enhanced signals. It can easily detect additional modes that cannot be observed in the traditional Raman spectrum. Therefore, SERS has been used for getting information of different modes presented in Gd‐doped CeO_2_ NPs.

**Figure 6:**
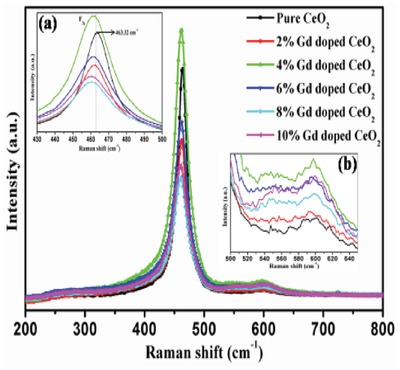
Raman spectra of pure CeO_2_ and Ce_1−*x*_Gd_*x*_O_2_ (*x* = 0.02, 0.04, 0.06, 0.08, and 0.10) nanoparticles. a,b) Inset of figure contains the enlarge views of their corresponding Raman spectra in the 430–500 cm^−1^ energy range related to F_2g_ mode and 500–650 cm^−1^ range related to oxygen defects, respectively.

**Figure** **6** shows Raman active (F_2g_) mode for pure CeO_2_ and Ce_1−*x*_Gd_*x*_O_2_ (*x* = 0.02, 0.04, 0.06, 0.08, and 0.10) samples, at 463.3 cm^−1^ and in the range of 461.4–459.5 cm^−1^, assigned for first‐order scattering.^56^ This Raman active mode is attributed to a symmetrical stretching mode of Ce‐O8 vibration unit. Therefore, this mode is very sensitive for any disorder in the oxygen sublattice results from nonstoichiometry of ceria.^57^ We can see from Figure 6 the absence of characteristic band for Gd_2_O_3_ (360 cm^−1^) clearly indicating the incorporation of Gd^3+^ ions into CeO_2_ lattice, confirms the absence of any impurity phase in the lattice, in agreement with XRD results.^58^, ^59^ F_2g_ mode corresponding to pure CeO_2_ and Ce_1−*x*_Gd_*x*_O_2_ is slightly shifted toward lower wavenumber side (or lower energy side) and broadening in its FWHM can also be observed with doping fluencies of Gd^3+^ ions in CeO_2_ sample. These structural changes in Raman spectra with Gd‐doping are attributed to the inhomogeneous strain and defects caused by substitution at the smaller radii Ce^4+^ (0.97 Å) site by larger ionic radii Gd^3+^ (1.08 Å) ions.^60^ In addition of F_2g_ mode, weak intensity second‐order Raman peaks are also obtained at 598.5 and 595.6 cm^−1^ for pure CeO_2_ and 2% Gd‐doped CeO_2_, respectively, generated due to nondegenerated longitudinal optical (LO) mode.^61^ These peaks are assigned to defect space that include intrinsic oxygen vacancies due to nonstoichiometry of CeO_2_. The three possible defect induced mechanism for oxygen vacancies in pure CeO_2_ sample can be given as^62^

(6) $${\mathrm{Ce}}_{\mathrm{Ce}}\;+\;2O_{O}\to V^{\prime \prime \prime \prime }+\;2V_{O}^{••}\;+\;{\mathrm{CeO}}_{2}$$

(7) $${\mathrm{Ce}}_{\mathrm{Ce}}\to V_{\mathrm{Ce}}^{\prime \prime \prime \prime }\;+\;{\mathrm{Ce}}_{i}^{••••}$$

(8) $$O_{O}\to V_{O}^{••}\;+\;O_{i}^{\prime \prime }$$

**Figure 7:**
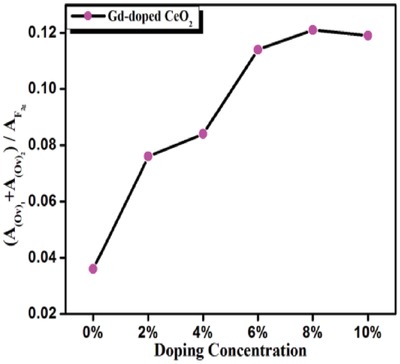
Relative peak area ratio for bands of oxygen vacancies and F_2g_ mode for Ce_1−*x*_Gd_*x*_O_2_ (*x* = 0.00, 0.02, 0.04, 0.06, 0.08, and 0.10) samples.

Since, these peaks are generated for maintaining the electrically neutrality in the system, therefore all Ce‐ions not only shows Ce^4+^ state but also Ce^3+^ state. For doing so, oxygen (O^2−^) ions are released from the structure and finally oxygen vacancies are formed in the system. The intensity of this peak is increased with incorporation of Gd‐ions indicates the rise of oxygen vacancies in ceria lattice. With increasing the fluency of Gd‐ions, two weak second‐order Raman modes in the range of 554.3–558 and 598–600.1 cm^−1^ are also obtained (as shown in the inset of Figure 6b). The Raman mode in the range of 554.3–558 cm^−1^ is related to the extrinsic oxygen vacancies, which are generated due to charge compensating defects due to substitution of Ce^4+^ ions by Gd^3+^ ions. The possible disorder mechanism for extrinsic oxygen vacancies in Ce_1−*x*_Gd_*x*_O_2_ (*x* = 0.02, 0.04, 0.06, 0.08, and 0.10) NPs can be given as^63^

(9) $${\mathrm{Gd}}_{2}O_{3}\;+\;2{\mathrm{Ce}}_{\mathrm{Ce}}^{x}+O_{O}^{x}\to 2{\mathrm{Gd}}_{\mathrm{Ce}}^{\prime }\;+\;V_{O}^{••}\;+\;{\mathrm{2CeO}}_{2}$$

where symbols have the following meaning as: ${\mathrm{Ce}}_{\mathrm{ce}}^{x}$ and ${\mathrm{Gd}}_{\mathrm{Ce}}^{\prime }$ are Ce^4+^ and Gd^3+^ ions on the CeO_2_ lattice site, respectively, $O_{O}^{x}$ is O^−2^ ions on an oxygen lattice site, and $V_{O}^{••}$ is neutral oxygen vacancy site. In addition, another vacancy peak can also be observed in the range of 598–600.1 cm^−1^, which is assigned to the defect space including intrinsic oxygen vacancies due to reduction of Ce^4+^ to Ce^3+^, i.e., nonstoichiometry of ceria.^62^ The possible disorder reaction for intrinsic oxygen vacancies in the sample can be given as^64^

(10) $$2{\mathrm{Ce}}_{\mathrm{Ce}}^{x}+\;O_{O}^{x}\to 2{\mathrm{Ce}}_{\mathrm{Ce}}^{\prime }\;+\;V_{O}^{••}\;+\;{\mathrm{1/2O}}_{2}\left(g\right)$$

where symbols have the following meaning as: ${\mathrm{Ce}}_{\mathrm{ce}}^{x}$ and ${\mathrm{Ce}}_{\mathrm{Ce}}^{\prime }$ are Ce^4+^ and Ce^3+^ ions on the Ce lattice site, respectively, $O_{O}^{x}$ is O^−2^ ions on an oxygen lattice site, and $V_{O}^{••}$ is neutral oxygen vacancy site.^65^ As shown in the inset of Figure 6b, the intensity of intrinsic and extrinsic oxygen vacancies mode increases with doping fluency up to *x* = 0.04. With further increase in fluency, the intensity of this mode decreases and then again increases at *x* = 0.10 concentration. The variation of intensity of vacancy mode is related to the concentration of oxygen vacancies. The quantitative estimation of oxygen vacancies of pure CeO_2_ and Gd‐doped CeO_2_ samples is made from the relative peak area of vacancy modes (intrinsic and extrinsic) with area of F_2g_ mode. For doing so, Lorentzian fitting is done for measuring the peak area of the respected peaks. All calculated values are tabulated in **Table** **2**, which indicates an increment in the concentration of oxygen vacancies with fluency of Gd‐ions up to *x* = 0.08 (i.e., maximum in this range) and then slightly decreased at *x* = 0.10 concentration. The increment in the concentration of oxygen vacancies can be explained by considering, with incorporation and rising fluency of large radii Gd^3+^ ions (0.105 nm), the dislocation density as well as strain has been increased up to *x* = 0.08 doping concentration in ceria. Due to this reason every two Gd^3+^ ions substituted the smaller radii Ce^4+^ (0.097 nm) ions increases the probability of oxygen ion (O^2−^) to leave the ceria lattice to maintain electrical neutrality in the lattice and creates more oxygen vacancies (as shown in **Figure** **7**).^68^ In addition to that, pure CeO_2_ and Gd‐doped CeO_2_ samples also exhibit one more extra weak second‐order Raman mode at 1064.9 cm^−1^ and in the range of 1173.9–1175.3 cm^−1^, (as shown in Table 2), which are assigned to 2LO mode that emanate from the second‐order scattering of the surface superoxide species $\left(O_{2}^{-}\right)$, and has small additional contribution from F_2g_ symmetry (which is not mentioned in Figure 6).^56^, ^65^

**Table 2:** The position of Raman active modes (cm^−1^) from Raman spectra and relative peak area ratio

Sample	Position of Raman active mode (cm^−1^) and vibrational mode					$A_{O_{v}}/A_{F_{2g}}$	$\left(A_{{\left(O_{V}\right)}_{1}}+A_{{\left(O_{V}\right)}_{2}}\right)/A_{F_{2g}}$
	First‐order scattering	Second‐order scattering^66^, ^67^					
	F_2g_	*A*_1g_ (O_v_)	*A*_1g_ + F_2g_ (O_v_)_1_	*A*_1g_ + *E*_g_ + F_2g_ (O_v_)_2_	2LO		
Pure CeO_2_	463.3	598.5	–	–	1064.9	0.036	–
Ce_0.98_Gd_0.02_O_2_	461.4	595.6	–	–	1173.9	0.076	–
Ce_0.96_Gd_0.04_O_2_	460.9	–	554.3	598	1175.6	–	0.084
Ce_0.94_Gd_0.06_O_2_	460.5	–	554.9	597	1176	–	0.114
Ce_0.92_Gd_0.08_O_2_	459.8	–	555.6	598.3	1175.7	–	0.121
Ce_0.90_Gd_0.10_O_2_	459.5	–	558	600.1	1175.3	–	0.119

Note: Vibrational modes corresponding to second‐order scattering were given based on refs. 66 and 67.

The particle size of all the Ce_1−*x*_Gd_*x*_O_2_ samples has been calculated from Raman spectra using the equation

(11) $$\Gamma \;\left({\mathrm{cm}}^{-1}\right)=\;10+\left(\frac{124.7}{D}\right)\;\mathrm{nm}$$

where Γ (cm^−1^) is full width at half maximum (FWHM) of Raman active (F_2g_) mode and *D* is particle size of pure CeO_2_ and Ce_1−*x*_Gd_*x*_O_2_ (*x* = 0.02, 0.04, 0.06, 0.08, and 0.10) samples.^61^, ^69^ The calculated particle size from Raman spectra is in good agreement with the particle size calculated from XRD and TEM images (as shown in Table 1). The quantitative estimation of the overall concentration of oxygen vacancies has been made by the peak area of the oxygen vacancies $A_{O_{v}}$ corresponding to 598.5 and 595.6 cm^−1^ for pure CeO_2_ and 2% Gd‐doped CeO_2_. The relative ratios of $A_{{\left(\mathrm{OV}\right)}_{1}}$, $A_{{\left(\mathrm{OV}\right)}_{2}}$ and $A_{F_{2g}}$ bands, which are corresponding to 554.3–558, 598–600.1, and for F_2g_ band is also calculated to estimate the oxygen vacancies concentration for further doping concentrations of Gd for 4% onward. The calculated values are shown in Figure 7.

This relative peak area ratio of oxygen vacancies and F_2g_ mode is calculated by fitting the Lorentzian function for the corresponding modes. Ratio $A_{O_{v}}/A_{F_{2g}}$ for pure CeO_2_ and 2% Gd‐doped CeO_2_, whereas, $(A_{{\left(\mathrm{OV}\right)}_{1}}\;+\;A_{{\left(\mathrm{OV}\right)}_{2}})/A_{F_{2g}}$ ratios are calculated for Ce_1−*x*_Gd_*x*_O_2_ (for *x* = 0.04, 0.06, 0.08, and 0.10) samples. From these calculated values one can infer that the relative oxygen vacancy concentration is found to gradually increase with fluencies of Gd^3+^ ions in CeO_2_ NPs.

### 2.5 X‐Ray Photoelectron Spectroscopy (XPS) Measurements

#### 2.5.1 XPS Spectra in Ce 3d Region

**Figure 8:**
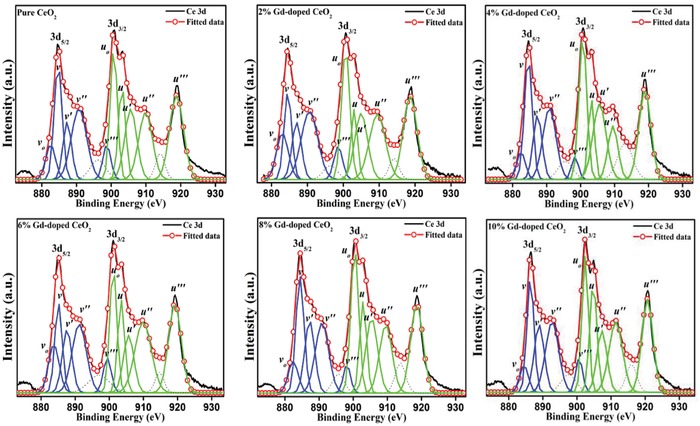
Deconvoluted XP spectra of Ce 3d profile of pure CeO_2_ and Ce_1−*x*_Gd_*x*_O_2_ (*x* = 0.02, 0.04, 0.06, 0.08, and 0.10) samples.

The chemical composition and the valence state of the pure CeO_2_ and Gd‐doped CeO_2_ NPs have been further characterized using XPS measurements of Ce 3d, Gd 4d, and O 1s core levels. **Figure** **8** illustrates the Ce 3d core level XP spectra of Ce_1−*x*_Gd_*x*_O_2_ (*x* = 0.02, 0.04, 0.06, 0.08, and 0.10) samples. All binding energies have been corrected for the charge shift using the C 1s peak (binding energy = 284.6 eV) as reference.^70^ The high‐resolution Ce 3d core level spectra in the energy range of 880–930 eV have been deconvoluted by mean of Gaussian shape fitting as shown in Figure 8. These deconvoluted Ce 3d core‐level spectra are generally characterized by distinct features which are related to the final‐state occupation of Ce 4f level.^71^ On Account of spin–orbit coupling, these deconvoluted Ce 3d core‐level spectra are resolved into ten peaks, which include six and four structures arise from Ce^4+^ and Ce^3+^ ions, respectively. These series of peaks are labeled as “*u*” and “*v*,” which are due to 3d_3/2_ and 3d_5/2_ spin–orbit states, respectively.^30^ The four peaks labeled with *v*_o_, *v*′, *u*_o_, and *u*′ are characteristic peaks of Ce^3+^, whereas, the peaks labeled with *v*, *v″*, *v‴*, *u*, *u″*, and *u‴* are characteristic peaks of Ce^4+^ (shown in Figure 8).^72^ The separation in binding energy between *v* and *u* spin–orbit doublets is found around ≈18.4 eV for pure CeO_2_, and for Ce_1−*x*_Gd_*x*_O_2_ (*x* = 0.02, 0.04, 0.06, 0.08, and 0.10) samples, which are in good agreement with the reported papers.^73^, ^74^

We can see from the Ce 3d core level spectra that Ce ions are present in mixed valance state of both Ce^3+^ and Ce^4+^ for pure CeO_2_ and Ce_1−*x*_Gd_*x*_O_2_ (*x* = 0.02, 0.04, 0.06, 0.08, and 0.10) samples. All peak positions for “*u*” and “*v*” of pure CeO_2_ and Ce_1−*x*_Gd_*x*_O_2_ (*x* = 0.02, 0.04, 0.06, 0.08, and 0.10) samples have been tabulated in **Table** **3**. The total concentration of Ce^3+^ and Ce^4+^ in the samples has been calculated using the following formula^75^

(12) $$\%\;{\mathrm{Ce}}^{3+}=\frac{A_{{\mathrm{Ce}}^{3+}}}{A_{{\mathrm{Ce}}^{3+}}+A_{{\mathrm{Ce}}^{4+}}}\;\times \;100\%$$

(13) $$\%\;{\mathrm{Ce}}^{4+}=\frac{A_{{\mathrm{Ce}}^{4+}}}{A_{{\mathrm{Ce}}^{3+}}+\;A_{{\mathrm{Ce}}^{4+}}}\;\times \;100\%$$

**Table 3:** Ce 3d XPS peak assignments for pure CeO_2_ and Ce_1−*x*_Gd_*x*_O_2_ (*x* = 0.02, 0.04, 0.06, 0.08, and 0.10) samples

Sample	Peak assignment	Ce 3d_5/2_					Ce 3d_3/2_					Ce^3+^ [%]	Ce^4+^ [%]	Ce^3+^/Ce^4+^
		*v*_o_ Ce^3+^	*v* Ce^4+^	*v′* Ce^3+^	*v″* Ce^4+^	*v‴* Ce^4+^	*u*_o_ Ce^3+^	*u* Ce^4+^	*u′* Ce^3+^	*u″* Ce^4+^	*u‴* Ce^4+^			
Pure CeO_2_	Binding energy [eV]	882.5	884.7	887.7	890.7	898.7	900.7	903.1	905.6	909.8	918.8	34.23	65.76	0.52
Ce_0.98_Gd_0.02_O_2_		883	884.5	887	890.5	898.5	900.6	903	904.9	909.5	918.6	38.86	61.13	0.63
Ce_0.96_Gd_0.04_O_2_		882.4	884.6	887.3	890.8	898.3	900.6	903.1	905.5	909.5	918.9	41.36	58.63	0.70
Ce_0.94_Gd_0.06_O_2_		883.7	885.1	887.6	891.2	899.3	901.1	903.4	905.6	909.9	919.2	35.04	64.95	0.54
Ce_0.92_Gd_0.08_O_2_		882.4	884.4	887.2	890.8	898.1	900.4	902.9	905.4	909.5	918.6	39.50	60.49	0.65
Ce_0.90_Gd_0.10_O_2_		884.4	886.3	888.9	892.7	900.6	902.4	904.7	907.3	911.3	920.6	33.61	66.38	0.51

Here, $A_{{\mathrm{Ce}}^{3+}}=v_{o}\;+\;v^{\prime }+\;u_{o}+\;u^{\prime }$ and $A_{{\mathrm{Ce}}^{4+}}=v\;+\;v^{\prime \prime }\;+\;v^{\prime \prime \prime }\;+\;u\;+\;u^{\prime \prime }\;+\;u^{\prime \prime \prime }$ are the sum of the integrated area of all characteristics peaks of Ce^3+^ and Ce^4+^, respectively. These calculated values are tabulated in Table 3. The quantitative ratio of Ce^3+^/Ce^4+^ shows that the concentration of Ce^3+^ ions over Ce^4+^ ions is gradually increasing for Ce_1−*x*_Gd_*x*_O_2_ (*x* = 0.00, 0.02, and 0.04) samples. While, at 6% Gd‐doping concentration Ce^3+^/Ce^4+^ value is deceased, which is again increased and then decreased at 8% and 10% doping.

This shows that due to incorporation of larger radii Gd^3+^ ions (0.105 nm) in CeO_2_ NPs, replacing the smaller radii Ce^4+^ ions (0.97 Å) and for maintaining the charge neutrality, the concentration of Ce^3+^ ions (0.114 nm) is gradually increased for *x* = 0.02 and 0.04 doping concentrations. The presence of Ce^3+^ may be due to either the formation of Ce_2_O_3_ or the creation of oxygen vacancies in CeO_2_ lattice. This can be verified by calculating the stoichiometry ratios *x* = [O]/[Ce] and *x*′ = [O_1s_]/[Ce_3d_], which can be estimated from their integrated peak area while considering their sensitivity factor. In order to calculate oxygen content in the samples, we assume that the total oxygen content is the sum of the required oxygen to fully oxidize Ce^3+^ and Ce^4+^ to form Ce_2_O_3_ and CeO_2_. Then, considering the stoichiometry *x* = [O]/[Ce], which is equal to 1.5 for Ce_2_O_3_ and 2 for CeO_2_. Now, the stoichiometric ratio of the oxygen to the total Ce ions (Ce^3+^ + Ce^4+^) can be determined using the concentration of [Ce^3+^] and [Ce^4+^] as given in **Table** **4** according to the following equation^52^

(14) $$x\;=\frac{\left[O\right]}{\left[\mathrm{Ce}\right]}\;=\;\frac{3}{2}\;\times \;\left[{\mathrm{Ce}}^{3+}\right]\;+2\;\times \;\left[{\mathrm{Ce}}^{4+}\right]$$

**Table 4:** Concentration of Ce^3+^ and Ce^4+^ ions and stoichiometry *x* = [O]/[Ce] and *x*′ = [O_1s_]/[Ce_3d_] of the pure CeO_2_ and Ce_1−*x*_Gd_*x*_O_2_ (*x* = 0.02, 0.04, 0.06, 0.08, and 0.10) samples

Sample	[Ce^3+^]	[Ce^4+^]	*x* = [O]/[Ce]a)	*x*′ = [O_1s_]/[Ce_3d_]b)
Pure CeO_2_	0.342	0.657	1.83	2.73
Ce_0.98_Gd_0.02_O_2_	0.388	0.611	1.80	1.84
Ce_0.96_Gd_0.04_O_2_	0.413	0.586	1.79	1.60
Ce_0.94_Gd_0.06_O_2_	0.350	0.649	1.82	1.71
Ce_0.92_Gd_0.08_O_2_	0.395	0.604	1.80	1.51
Ce_0.90_Gd_0.10_O_2_	0.336	0.663	1.83	1.61

^a)^ Using Equation (14)

^b)^ Using Equation (15).

**Figure 9:**
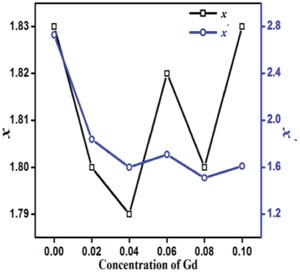
The CeO*_x_* stoichiometry for pure CeO_2_ and Gd‐doped CeO_2_ samples calculated from stoichiometry ratio *x* = [O]/[Ce] and *x*′ = [O_1s_]/[Ce_3d_].

The stoichiometry calculated from Equation (14) has been compared with the actual stoichiometry determined from the XPS integrated area *A*_O_ and *A*_Ce_ of the O 1s and Ce 3d peaks, respectively, which has been calculated according to the following equation^76^

(15) $$x^{\prime }=\frac{O_{1s}}{{\mathrm{Ce}}_{3d}}\;=\frac{A_{O}}{A_{\mathrm{Ce}}}\;\times \;\frac{S_{\mathrm{Ce}}}{S_{O}}$$

where *S*_Ce_ = 7.399 and *S*_O_ = 0.711 are the sensitivity factors of the Ce and O atoms, respectively.^77^ **Figure** **9** shows the stoichiometry variation with the concentration of Gd‐dopant determined by both methods, *x* and *x′*, which is provided the concentration of Ce^3+^ and Ce^4+^ ions in pure CeO_2_ and Ce_1−*x*_Gd_*x*_O_2_ (*x* = 0.02, 0.04, 0.06, 0.08 and 0.10) samples (as listed in Table 4).

Although, the calculated values of actual stoichiometry (*x′*) are higher than that (*x*) calculated by Equation (5) for pure CeO_2_ and Ce_0.98_Gd_0.02_O_2_ sample, which exhibits low concentration of Ce^3+^ ions for pure CeO_2_ NPs in comparison with Ce_0.98_Gd_0.02_O_2_ sample. This means that due to incorporation of Gd^3+^ ions in CeO_2_ NPs, Gd^3+^ ions replaces the Ce^4+^ ions with formation of oxygen vacancies in the Ce_0.98_Gd_0.02_O_2_ sample. On the other hand, the value of (*x′*) is smaller than (*x*) for *x* = 0.04, 0.06, 0.08, and 0.10 doping concentrations, which suggested that the entire Ce^3+^ ions are consumed in the formation of Ce_2_O_3_. Simultaneously, the oxygen deficiency with increasing Ce^3+^ ions suggests that Ce^3+^ ions are associated with Ce_2_O_3_ as well as oxygen vacancies in CeO_2_ and both kinds may coexist in Gd‐doped CeO_2_ (*x* = 0.04, 0.06, 0.08, and 0.10) samples.

This means that core level Ce 3d spectra prove the existence of Ce_2_O_3_ in the Ce_1−*x*_Gd_*x*_O_2_ (*x* = 0.02, 0.04, 0.06, 0.08, and 0.10) samples, while from XRD analysis only CeO_2_ is identified. This Ce_2_O_3_ phase has amorphous character and indicates that this phase is located at the grain surface and at the grain boundaries. Patsalas et al.^52^ have reported a dimensional analysis, which determined that Ce_2_O_3_ and CeO_2_ are located at grain surface and volume, respectively. A linear correlation can be established between third power of [Ce^4+^] (grain volume distribution) as well as third power of *D* (which is proportional to the grain volume *V*_g_) with square of [Ce^3+^] (surface distribution).

**Figure 10:**
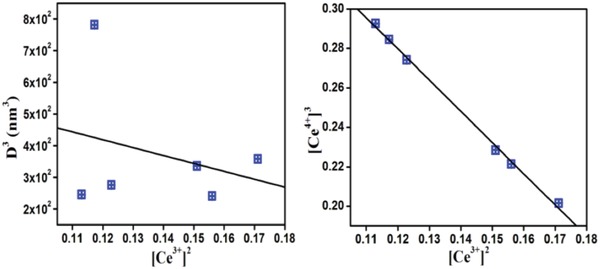
The correlation of the [Ce^3+^]^2^ with [Ce^4+^]^3^ and grain volume (*V*_g_ ∝ *D* ^3^), showing that Ce^3+^ and Ce^4+^ ions are located at the grain surface and volume, respectively.

**Figure** **10** shows a linear relation between [Ce^3+^]^2^ and [Ce^4+^]^3^ that confirms the distribution of Ce_2_O_3_ and CeO_2_ at the grain surface and volume. While, the experimental points of *D* ^3^ versus [Ce^3+^]^2^ are more scattered around straight line of the dimensional analysis, which is attributed to the strain in the grain that affects the broadening of the XRD peaks with Gd‐doping in CeO_2_ samples. From Table 4, it can also be seen that the difference between *x* and *x′* increases and decreases with increase and decrease of Ce^3+^ ions for Ce_1−*x*_Gd_*x*_O_2_ (*x* = 0.04, 0.06, 0.08, and 0.10) samples, which show an up and down in the formation of oxygen vacancies in these samples.

#### 2.5.2 O 1s XPS Spectra

**Figure 11:**
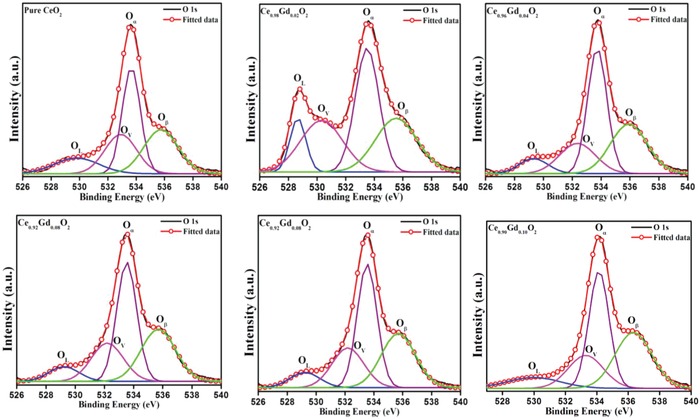
Deconvoluted core level spectra of O 1s profile for pure CeO_2_ and Ce_1−*x*_Gd_*x*_O_2_ (*x* = 0.02, 0.04, 0.06, 0.08, and 0.10) samples.

The O 1s spectra for pure CeO_2_ and Ce_1−*x*_Gd_*x*_O_2_ (*x* = 0.02, 0.04, 0.06, 0.08, and 0.10) samples are shown in **Figure** **11**. The asymmetrical O 1s core level spectra in the binding energy range 526–540 eV are deconvoluted into four peaks to determine the surface concentration of oxygen ions for all samples. The deconvoluted binding energy peaks of O 1s core level spectra at ≈528.6–529.9 eV can be assigned to the lattice oxygen O^2−^ (denoted as O_L_) in pure CeO_2_ and Ce_1−*x*_Gd_*x*_O_2_ (*x* = 0.02, 0.04, 0.06, 0.08, and 0.10) samples, while peak at higher binding energy side ≈530.3–533.3 eV and ≈533.5–536.4 eV are possibly assigned to oxygen vacancies (denoted as O_V_) corresponds to Ce^3+^ species originated from Ce_2_O_3_ 78 and formation of hydroxyl or absorbed H_2_O species^79^ (denoted as O_α_ and O_β_) on the surface of the samples, respectively (as shown in **Table** **5**).^80^, ^81^

**Table 5:** XPS binding energies of individual peaks of O 1s spectra for pure CeO_2_ and Ce_1−*x*_Gd_*x*_O_2_ (*x* = 0.02, 0.04, 0.06, 0.08, and 0.10) samples

Sample	O 1s peak position					
	Lattice oxygen species (O_L_)		Oxygen vacancy species (O_V_)		OH ^−^ group species BE [eV]	
	BE [eV]	$\%\;O_{L}=\frac{A_{O_{L}}}{A_{O_{L}}+A_{O_{V}}}\;\times \;100$	BE [eV]	$\%\;O_{V}=\frac{A_{O_{V}}}{A_{O_{L}}+A_{O_{V}}}\;\times \;100$	(O_α_)	(O_β_)
Pure CeO_2_	529.8	35.94	532.9	64.05	533.6	535.7
Ce_0.98_Gd_0.02_O_2_	528.6	28.16	530.3	71.84	533.5	535.5
Ce_0.96_Gd_0.04_O_2_	529.3	28.41	532.4	71.58	533.7	535.9
Ce_0.94_Gd_0.06_O_2_	529.8	35.79	532.6	64.20	533.9	536.1
Ce_0.92_Gd_0.08_O_2_	529.2	26.60	532.2	73.40	533.5	535.7
Ce_0.90_Gd_0.10_O_2_	529.9	33.32	533.3	66.70	534.1	536.3

As shown in Figure 11, all the samples are showing the similar O 1s core level spectra, which are also used as another source of information about Ce oxidation state. Since, it is well known that the electronegativity of Gd ion (1.21) is higher than Ce ion (1.12) on Pauling scale, therefore, O 1s peak from Gd_2_O_3_ should be at higher binding energy than that from metal oxide CeO_2_.^82^ Thus, due to incorporation of Gd^3+^ ions in the lattice of CeO_2_, not only the intensity of the lattice oxygen peak (O_L_) but also oxygen vacancies peak (O_V_) are found to increase for 2% Gd‐doped CeO_2_ NPs. The quantitative estimation of O_L_ and O_V_ peaks shows that due to incorporation of Gd^3+^ ions the oxygen vacancies are formed on the surface of the Gd‐doped CeO_2_ samples. These vacancies are found to show variation with change in the concentration of Gd‐ions in the CeO_2_ NPs. Furthermore, as 1s electron of oxygen atom attached more tightly bound to Ce^3+^ rather than Ce^4+^ oxidation state. Thus, change in the oxidation state of Ce‐ions (+4 to +3) due to incorporation of Gd^3+^ ions in the CeO_2_ NPs, may also be responsible for the change in the formation of oxygen vacancies. The quantitative percentage of Ce^3+^ oxidation state from core level spectra of Ce 3d for pure CeO_2_ and Ce_1−*x*_Gd_*x*_O_2_ (*x* = 0.02, 0.04, 0.06, 0.08, and 0.10) samples, if compared with quantitative percentage of O_V_ from O 1s core level spectra, one can infer that the increasing Ce^3+^ concentration is also helpful in increasing the oxygen vacancies on the surface of samples (as shown in Tables 3 and 5) along with the percentage increase in the concentration of the Gd^3+^ ions.

#### 2.5.3 Gd 4d XPS Spectra

**Figure 12:**
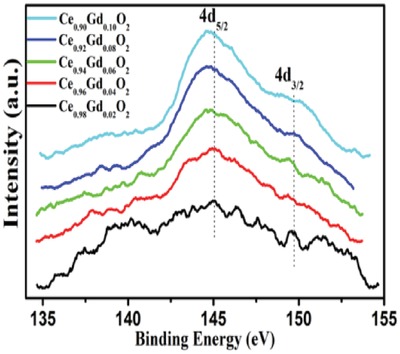
Gd 4d core level XP spectra of Ce_1−*x*_Gd_*x*_O_2_ (*x* = 0.02, 0.04, 0.06, 0.08, and 0.10) samples.

The deconvoluted Gd 4d core level XPS spectra are split into doublet (Gd 4d_5/2_ and Gd 4d_3/2_) due to spin–orbit coupling for Ce_1−*x*_Gd_*x*_O_2_ (*x* = 0.02, 0.04, 0.06, 0.08, and 0.10) samples, as shown in **Figure** **12**. These two peaks existed in the range of ≈143.7–145.8 eV and ≈148.7–151.7 eV can be attributed to Gd 4d_5/2_ and Gd 4d_3/2_ states, respectively, which are indicating the presence of Gd^3+^ ions in Ce_1−*x*_Gd_*x*_O_2_ (*x* = 0.02, 0.04, 0.06, 0.08, and 0.10) doped lattice.^83^, ^84^, ^85^

### 2.6 Magnetic Measurements

**Figure 13:**
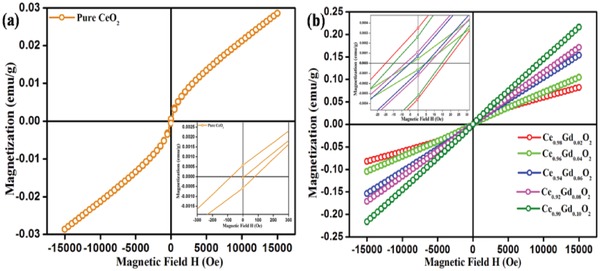
Magnetization versus magnetic field plot for a) pure CeO_2_ and b) Ce_1−*x*_Gd_*x*_O_2_ (*x* = 0.02, 0.04, 0.06, 0.08, and 0.10) samples at room temperature (300 K).

**Figure** **13**a,b shows the room temperature magnetization (*M*) versus magnetic field (*H*) curves for pure CeO_2_ and Ce_1−*x*_Gd_*x*_O_2_ (*x* = 0.02, 0.04, 0.06, 0.08, and 0.10) NPs. It is observed that pure CeO_2_ nanoparticles are found to exhibit weak ferromagnetic (FM) behavior at room temperature with saturation magnetization *M*_s_ = 0.049 emu g^−1^. Although, it has been reported that bulk CeO_2_ exhibit diamagnetic behavior where it is reported that at nanoregime the undoped CeO_2_ NPs exhibit weak ferromagnetism with small value of saturation magnetization by few reports.^86^, ^87^, ^88^, ^89^, ^90^, ^91^, ^92^ Since, a significant amount of coercivity *H*_c_ = 77.95 Oe has been observed for pure CeO_2_ NPs, which ensures the ferromagnetic nature in our pure CeO_2_ sample. The weak ferromagnetic behavior in pure CeO_2_ NPs at room temperature is associated with oxygen vacancies that have been originated by the conversion of Ce^4+^ to Ce^3+^ oxidation state of cerium.^93^

Although, after incorporation of Gd^3+^ ions in CeO_2_ NPs, Ce_0.98_Gd_0.02_O_2_ sample still exhibit weak ferromagnetic (FM) behavior with increasing *M*_s_ = 0.140 emu g^−1^ while *H*_c_ has been decreased 22.48 Oe as compared to pure CeO_2_ NPs. While, further increase in Gd^3+^ ions concentration are not able to maintain this FM behavior, that can be clearly seen from the hysteresis curves (in Figure 13b) for Ce_1−*x*_Gd_*x*_O_2_ (*x* = 0.04, 0.06, 0.08, and 0.10) samples. The magnetization of the Gd‐doped CeO_2_ samples is increased with increasing dopant concentration (as shown in **Table** **6** and Figure 13b). Since, the electronic configuration of Gd^3+^ is [Xe] 6s^2^5d^1^4f^7^ with 7 unpaired electrons in the 4f shell. These unpaired 4f electrons polarize the 6s and 5d valence electrons, results high effective magnetic moment *µ*_eff_ = 7.94 *µ*_B_ (calculated by the formula $\mu_{\mathrm{eff}}\;=\;g_{J}\sqrt{J(J+1)}\;\mu_{B}$, where *g_J_* is the Lande *g*‐factor and for Gd^3+^ ion ground state ^8^ *S*_7/2_, *S* = 7/2, *L* = 0, *J* = 7/2, *g_J_* = 2).^94^, ^95^ With increasing dopant concentration, the interaction of these unpaired spins of 4f electrons with the outermost ligands or other Gd^3+^ is anticipated to get weaker. These noninteracting and localized magnetic spins of Gd^3+^ ions have induced the paramagnetism with increase in magnetization.^96^, ^97^ The paramagnetic moment from the Gd^3+^ ions incorporated into the CeO_2_ lattice increases with increasing the dopant concentration, which results in reduction of ferromagnetic ordering in Gd‐doped samples. Therefore, 4%, 6%, 8%, and 10% Gd‐doped samples have small ferromagnetic behavior in addition to linear paramagnetic signals, which is gradually increasing with the fluency of Gd^3+^ ions in CeO_2_ NPs. Though Raman and XPS analyses are showing an increase in the oxygen vacancies but this increase in oxygen vacancy concentration may not enhance the ferromagnetic ordering in Gd‐doped samples.^98^ Nithyaa and Jaya reported the ferromagnetic behavior of pure TiO_2_ NPs but incorporation of Gd‐ions enhanced the paramagnetic nature, which has been reported due to oxygen defects.^99^ In other reports on Gd doping in ZnO, the paramagnetism in these samples is reported due to high magnetic moment of Gd‐ions (μ = 7.1 μ_B_) and due to presence of secondary phases of Gd_2_O_3_.^100^, ^101^, ^102^

**Table 6:** Summary of saturation magnetization (*M*_s_), retentivity (*M*_r_), and coercivity (*H*_c_) for pure CeO_2_ and Ce_1−*x*_Gd_*x*_O_2_ (*x* = 0.02, 0.04, 0.06, 0.08, and 0.10) NPs

Sample	*M*_s_ [emu g^−1^]	*M*_r_ [emu g^−1^] [× 10^−4^]	*H*_c_ [Oe]
Pure CeO_2_	0.049	5.77	77.95
Ce_0.98_Gd_0.02_O_2_	0.140	3.56	22.48
Ce_0.96_Gd_0.04_O_2_	0.194	0.46	1.30
Ce_0.94_Gd_0.06_O_2_	0.296	0.87	1.38
Ce_0.92_Gd_0.08_O_2_	0.333	1.16	6.18
Ce_0.90_Gd_0.10_O_2_	0.421	3.15	12.96

Now, the main issue herein is to understand the possible origin of ferromagnetic dominated paramagnetic behavior in pure CeO_2_ and Gd‐doped CeO_2_ NPs, respectively. The origin of FM behavior has been discussed in this paper accounting the F‐center exchange (FCE) mechanism as a subcategory of bound magnetic polaron (BMP) model.^103^ The conception of FCE coupling is based on BMP model that has been interpreted with the presence of oxygen vacancies (*V*_O_). These oxygen vacancies and magnetic ions constitute a BMP that produces the ferromagnetism in these systems. In pure CeO_2_ NPs, the origin of ferromagnetism is supposed to the reduction of the oxidation state of Ce ions, i.e., Ce^4+^ to Ce^3+^. The formations of oxygen vacancies give rise to the reduction of Ce^4+^ to Ce^3+^ state. The formation of oxygen vacancy left two electrons which may be transferred to a Ce^4+^ ion converting Ce^4+^ into Ce^3+^. Due to this process, mixed Ce^3+^ and Ce^4+^ states yield in the pure CeO_2_ NPs, which has already been confirmed by Ce 3d core level spectra analysis. The ferromagnetism in pure CeO_2_ NPs may be arise from the nearest‐neighbor interaction, i.e., either double exchange (Ce^3+^–*V*_O_–Ce^4+^) or superexchange (Ce^3+^–*V*_O_–Ce^3+^), which is mediated by oxygen ions.^104^ The double exchange interaction forms an F^+^ center because the two electrons left by *V*_O_ are trapped on Ce^4+^ ion and *V*_O_ (hydrogenic orbital), while superexchange interaction forms an F^2+^ center due to the both electrons are trapped on Ce^4+^ ions.^105^ When Gd ion is incorporated into CeO_2_, it has suppressed the ferromagnetism of CeO_2_ NPs (as shown in Figure 13b). Now, for Ce_0.98_Gd_0.02_O_2_ sample, the F^+^ center may be coupled with the nearest Ce^3+^ or Gd 4f orbital and form Ce^3+^–*V*_O_–Gd^3+^ complex (BMP), which is dominated in this sample. When the size of this BMP is large enough to percolate through the lattice, long‐range (weak) room temperature ferromagnetism can be realized with higher saturation magnetization. However, it is clearly observed that ferromagnetism has been suppressed with the increase in Gd‐doping concentration up to *x* = 0.10. Due to increase in Gd‐ion doping concentration, the number of Gd‐ions in the interior of CeO_2_ lattice is less than that on its surface or on the grain boundaries. Only those Gd‐ions are allowed to enter the lattice that is permitted by the host ions and rest is expelled. Due to higher doping concentration the separation among Gd^3+^ ions is decreased. These largely separated Gd^3+^ ions suppress the ferromagnetism and undergo superexchange interaction with each other via O^2−^ ions and results in antiparallel alignment of the magnetic spins of Gd 4f shell present in the nearest‐neighbor ions that do not negotiate in ferromagnetic ordering. Thus higher doping concentration of Gd^3+^ ions tends to destroy the observed ferromagnetism in Gd‐doped CeO_2_ NPs. Hence in our case the increase in paramagnetic signals may be attributed to increase in oxygen vacancy concentrations without enhancing the ferromagnetic ordering of the samples. This ferromagnetic ordering is further suppressed due to the increased concentrations of Gd^3+^‐cation as the separation between these Gd^3+^ ‐ions is decreased results in anti‐parallel alignment of the spins of Gd 4f state due to superexchange interaction.

### 2.7 Water Splitting Analysis

**Figure 14:**
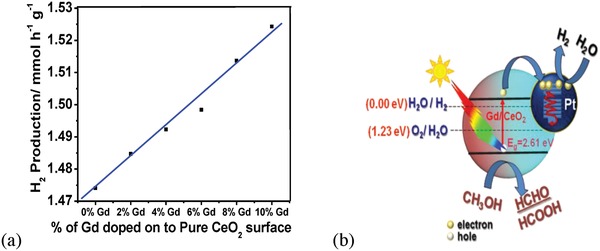
a) Hydrogen production rate for pristine CeO_2_ and 2%, 4%, 6%, 8%, and 10% Gd‐doped CeO_2_ compounds in 10% CH_3_OH under visible light exposure of 300 W Xe light source and b) charge transfer reaction at oxidative and reductive sites.

The amount of photocatalytic H_2_ evolved from the samples has been hourly monitored (**Table** **7** and **Figure** **14**a) and after 4 h exposure to light the respective release of hydrogen is observed as 1.47406, 1.4847, 1.4923, 1.4984, 1.51367, and 1.5243 mmol h^−1^ g^−1^ for pristine Pt/CeO_2_, Pt/Gd_0.02_Ce_0.98_O_2_, Pt/Gd_0.04_Ce_0.96_O_2_, Pt/Gd_0.06_Ce_0.94_O_2_, Pt/Gd_0.08_Ce_0.092_O_2_, Pt/Gd_0.10_Ce_0.90_O_2_ samples, respectively (Figure 14a). According to the mechanism, when the surface of the molecular device Pt/GdCeO_2_ exposed to the light, an electron of the valance band (VB) gets energized after receiving that energy of light and jumped from VB to conduction band (CB), which generates a pair of photohole (at VB) and photoelectron (at CB) at Gd/CeO_2_ surface. Nascent photoelectrons of CB are arrived at the junction of Pt/electrolyte interface by passing through the electron‐pool of the metallic Pt (that can segregate the photoelectrons from photoholes). These photoelectrons interact with H^+^ ions^106^ of the water at the interface and liberate the nascent H that combined with another nascent H atom to generate H_2_ gas. Hole amassed at VB of the doped semiconductor is responsible for the breakdown of CH_3_OH in formaldehyde or formic acid or both as mentioned in Equations (16)–(26),^107^, ^108^ which can be used to depict the proposed electron transfer mechanism of the water splitting, as illustrated by Figure 14b.

(16) $$\begin{array}{l} \mathrm{Gd}-{\mathrm{CeO}}_{2}\;\overset{\mathrm{light}}{\to }\left(\mathrm{CV}\;\mathrm{of}\;\mathrm{Gd}-{\mathrm{CeO}}_{2}\right)\;e^{-}\\ \;+\left(\mathrm{VB}\;\mathrm{of}\;\mathrm{Gd}-{\mathrm{CeO}}_{2}\right)\;h^{+}\;\left(\mathrm{photocarriers}\;\mathrm{generation}\right) \end{array}$$

(17) (CV of Gd−CeO2)e−→Pt e− (transfer of photoelectron)

(18) $$\begin{array}{l} (\mathrm{CV}\;\mathrm{of}\;\mathrm{Gd}-{\mathrm{CeO}}_{2})e^{-}+H_{2}O\to H^{*}\\ \;\;\;+{\mathrm{OH}}^{-}\;(\mathrm{generation}\;\mathrm{of}\;\mathrm{free}\;\mathrm{radical}\;H^{*}) \end{array}$$

(19) $$H^{*}+H^{*}\to H_{2}(0.00\;\mathrm{eV})\;(\mathrm{generation}\;\mathrm{of}\;H_{2})$$

(20) $$H^{*}+\;h^{+}\to H^{+}(\mathrm{generation}\;\mathrm{of}\;H^{+})$$

(21) $$H^{+}+H^{+}\to H_{2}(0.00\;\mathrm{eV})\;(\mathrm{generation}\;\mathrm{of}\;H_{2})$$

(22) $$\left(\mathrm{VB}\;\mathrm{of}\;\mathrm{Gd}-{\mathrm{CeO}}_{2}\right)\;h^{+}+{\mathrm{CH}}_{3}\mathrm{OH}\to \;{\mathrm{CH}}_{2}{\mathrm{OH}}^{*}+H^{+}\;\left(-0.1264\;\mathrm{eV}\right)$$

(23) $$\begin{array}{l} h^{+}+{\mathrm{CH}}_{2}{\mathrm{OH}}^{*}+{\mathrm{OH}}^{-}\to {\mathrm{HOCH}}_{2}{\mathrm{OH}}^{*}\to \mathrm{HCHO}\\ \;+H_{2}O\;(-3.848\;\mathrm{eV})\;(\mathrm{formation}\;\mathrm{of}\;\mathrm{HCHO}) \end{array}$$

(24) $$\begin{array}{l} {\mathrm{CH}}_{2}{\mathrm{OH}}^{*}+O\to {\mathrm{OCH}}_{2}{\mathrm{OH}}^{*}\to \mathrm{HCHO}\\ \;+{\mathrm{OH}}^{*}(-3.18\;\mathrm{eV})\;(\mathrm{formation}\;\mathrm{of}\;\mathrm{HCHO}) \end{array}$$

(25) $$\begin{array}{l} {\mathrm{CH}}_{2}{\mathrm{OH}}^{*}+O\to {\mathrm{OCH}}_{2}{\mathrm{OH}}^{*}\to \mathrm{HCOOH}\\ \;+H^{*}\;(-4.125\;\mathrm{eV})\;(\mathrm{formation}\;\mathrm{of}\;\mathrm{HCOOH}) \end{array}$$

(26) $$\begin{array}{l} \mathrm{HCOOH}+2h^{+}\to {\mathrm{CO}}_{2}\\ \;+2H^{+}(-0.19\;\mathrm{eV})\;(\mathrm{formation}\;\mathrm{of}\;{\mathrm{CO}}_{2}\;\mathrm{from}\;\mathrm{HCHO}) \end{array}$$

**Table 7:** Comparative band gaps with their CB and VB positions, hydrogen production with and without Pt loading, with respect to the pure CeO_2_ and 2%, 4%, 6%, 8%, and 10% Gd‐doped CeO_2_ compound

Compound	Oxygen vacancy [%]	〈*D*〉 [nm]	Ce^3±^/Ce^4+^	Band gap [eV]	CB [eV]	VB [eV]	H_2_ generation without Pt loading [mol g^−1^ h^−1^]	H_2_ generation with Pt loading [mol g^−1^ h^−1^]
Pure CeO_2_	64.05	8.50	0.52	2.60	−0.240	2.360	(1.4695)	1.47406
Ce_0.98_Gd_0.02_O_2_	71.84	6.58	0.63	2.66	−0.270	2.390	(1.4771)	1.4847
Ce_0.96_Gd_0.04_O_2_	71.58	6.66	0.70	2.71	−0.295	2.415	(1.4832)	1.4923
Ce_0.94_Gd_0.06_O_2_	64.20	6.68	0.54	2.67	−0.275	2.395	(1.4923)	1.4984
Ce_0.92_Gd_0.08_O_2_	73.40	5.81	0.65	2.64	−0.260	2.380	(1.5030)	1.51367
Ce_0.90_Gd_0.10_O_2_	66.70	5.88	0.51	2.52	−0.200	2.320	(1.5167)	1.5243

There are many factors which can dominate the water splitting activity such as particle size of photocatalyst, binding energy, dopant concentration and position (either Gd^3+^ ion is taking position of Ce^3+^ or Ce^4+^ ion), oxygen vacancies, band gap and band positions, and many more. All of the above factors collectively responsible for increase in hydrogen generation activity on increasing the dopant concentration in ceria. Usually the steady decrease in particle size increases water splitting activity with increase in Gd proportion due to the large. Introduction of dopant Gd into the CeO_2_ lattice, also gradually increases the oxygen vacancy in the lattice arrangement of CeO_2_ because Gd^3+^(radius of Gd^3+^ = 0.105 nm and charge density = 91) replaced the high charged but small Ce^4+^ ion(radius of Ce^4+^ = 0.097 nm and charge density = 148) in 2%, 4%, and 8% doped samples but also replaced low charged but bigger sized Ce^3+^ (radius of Ce^3+^ cation = 0.114 nm and charge density = 75) in 6% and 10% Gd samples.^109^

**Figure 15:**
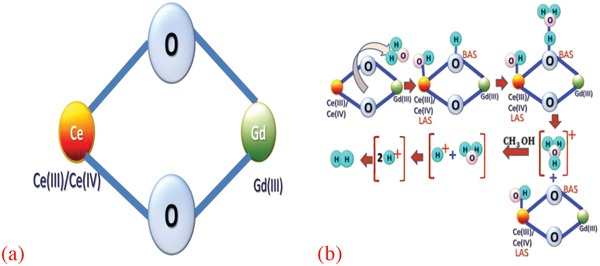
a) M–M bond through bridging O atoms and b) water splitting phenomena at atomic lattice level through Lewis acid site (LAS) and Brønsted acid site (BAS).

All of the above changes due to Gd‐doping in ceria lattice maintained the phase purity (checked with XRD) with minor but favorable changes in lattice parameters and suggested the lattice arrangement of atoms with expanded electron clouds between high charge M(Ce^4+^/Ce^3+^) and low charge M(Gd^3+^) bonds through bridging O and O as shown in **Figure** **15**a. That results into creating active side to generate more carriers that bring about the enhanced photocatalytic activity of the doped ceria.

Usually the steady decrease in particle size increases water splitting activity with increase in Gd proportion due to the large. Introduction of dopant Gd into the CeO_2_ lattice also gradually increases the oxygen vacancy in the lattice arrangement of CeO_2_ because Gd^3+^ (radius of Gd^3+^ = 0.105 nm and charge density = 91) replaced the high charged but small Ce^4+^ ion (radius of Ce^4+^ = 0.097 nm and charge density = 148) in 2%, 4%, and 8% doped samples but also replaced low charged but bigger sized Ce^3+^ (radius of Ce^3+^ cation = 0.114 nm and charge density = 75) in 6% and 10% Gd samples.^109^

Local cluster framework of the tetrahedral coordinated groups of multivalent metal cations (Ce^4+^/Ce^3+^and Gd^3+^) and anions ($O_{2}^{2-}$) generates a strong local electrostatic field inside the tetrahedra, as confirmed by the XRD, XPS, and Raman results. Residual water molecules are captured by the strong local electrostatic field of the molecular device Ce_1−*x*_Gd_*x*_O_2_ (*x* = 0.02, 0.04, 0.06, 0.08, and 0.10). These water molecules attract the bridging oxygen through the protonic side and the metallic cation, i.e., Ce through the hydroxyl side (Figure 15b).

Finally, we get bridging oxygen impregnated with a hydroxyl proton and the Ce metallic side with a hydroxyl group that function as Lewis acid sites, which create strong electron withdrawing centers neighboring bridging O–H groups^110^ as shown by Figure 15b. These withdrawing centers can act as superacidic Brnsted acid sites (BAS) with a highly negative cluster framework. H_3_O^+^ that detached from BAS to release the tension of the bulky species and generate H^+^. These H ^+^ reacts with the photoelectrons of the solid solutions and produce nascent H that couples with another H. Thus, hydrogen gas is generated.

## 3 Conclusion

In summary, Gd‐doped CeO_2_ nanoparticles have been successfully synthesized by the coprecipitation method. The structural and morphological studies have been made by XRD, TEM, HRTEM, and SEAD analysis. From the XRD analysis all the lattice parameters, volume, particle size of pure and Gd‐doped CeO_2_ NPs have been calculated, the particle size of these nanoparticles is further verified with TEM and SERS analysis and observed approximately similar to the results obtained with XRD analysis. The particles are spherical in shape and getting agglomerated with fluencies of Gd^3+^ ions in CeO_2_ sample and the particle size is in the range of 5–7 nm, which is confirmed from the TEM images. From the TEM results and analysis we have observed the broadening of diffraction rings, which indicates that the particles are small in size and crystallinity becomes low with increasing doping concentration of Gd^3+^ ions in CeO_2_ NPs.

From the analysis of the UV‐Vis‐NIR absorption spectra we have observed the variation in the refractive index and band gap energy with different concentration of Gd‐ions in CeO_2_ NPs. The reduction of refractive index with incorporation of Gd^3+^ ions in CeO_2_ NPs is beneficial to UV protection. Normally, UV protection ability is strongly depending on the particle size and at nanoregime UV absorption ability is stronger than that of microsized ones. From the SERS spectra on pure and Gd‐doped CeO_2_ NPs, the particle size, oxygen vacancy concentrations, etc. have been made to understand the mechanism of other properties of these samples. From the SERS spectra we can say that no other impurity phases are present in our samples and hence the nature of ferromagnetism in these NPs is intrinsic in nature and derived from the defects and intrinsic and extrinsic oxygen vacancy concentrations, which is found to gradually increase with fluencies of Gd^3+^ ions in CeO_2_ NPs.

Further for electronic structure of these NPs the core level Ce 3d, O1s, and Gd 4d XPS spectra have been recorded and analyzed in detail. From this analysis the change of oxidation state of Ce^+4^ to Ce^+3^ ions, incorporation of the Gd^+3^ ions in the lattice and formation of oxygen vacancies are reported. From the core level Ce 3d spectra the presence of both Ce^3+^ and Ce^4+^ ions in all the samples and an increase in the Ce^3+^ concentration can be seen with the fluencies of Gd^3+^ dopant ions in CeO_2_ NPs, due to the formation of defects or an amorphous phase of Ce_2_O_3_. From the O 1s core level spectra analysis we are able to demonstrate that doping of Gd^3+^ ions in CeO_2_ NPs can modify the rate of reduction of Ce^4+^ to Ce^3+^ oxidation state as well as affects the formation of oxygen vacancies in Ce_1−*x*_Gd_*x*_O_2_ (*x* = 0.02, 0.04, 0.06, 0.08, and 0.10) doped samples. However, the Gd 4d core level spectra cannot be recorded with good statistics due to very small concentrations but reports the presence of Gd ion in our NPs. In our magnetic measurements we can see that all the samples pure CeO_2_ and Ce_1−*x*_Gd_*x*_O_2_ (*x* = 0.02, 0.04, 0.06, 0.08, and 0.10) are showing the weak ferromagnetism dominated paramagnetic behavior, which is assumed to be triggered due to change of oxidation state of Ce^+4^ to Ce^+3^ ions, incorporation of the Gd^+3^ ions in the lattice and formation of oxygen vacancies. From Raman and XPS analyses, the presence of oxygen vacancy related defects is apparent. From Ce 3d XPS spectra one can reveal that a significant increase in Ce^3+^ ions is not only able to increase the oxygen vacancies due to formation of Ce_2_O_3_ phase in Ce_1−*x*_Gd_*x*_O_2_ (*x* = 0.02, 0.04, 0.06, 0.08, and 0.10), which is not traceable with the XRD analysis but also may be due to the substitution of Ce^4+^ ions by Gd^3+^ ions and this substitution actually creates the oxygen vacancies and owe the presence of ferromagnetic ordering in Ce_0.98_Gd_0.02_O_2_ sample.

In the water splitting results, the amount of photocatalytic H_2_ evolved from the samples is monitored, and the respective release of hydrogen is found to increase for pristine Pt/CeO_2_, Pt/Gd‐doped CeO_2_ samples with the increased doping concentrations of the Gd‐ion. The particle size of photocatalyst, binding energies, oxygen vacancy concentrations, band gap, and many other factors are collectively responsible for increase in hydrogen generation activity with increasing dopant concentration in ceria. The observed release of hydrogen is found in good correlation with the characterization results and the proposed mechanism of water splitting is reported on the basis of analyses.

## 4 Experimental Section

*Materials*: Ammonium cerium (IV) nitrate (NH_4_)_2_Ce(NO_3_)_6_ (Alpha Aesar 99.99%), gadolinium (III) nitrate hexahydrate Gd(NO_3_)_3_·6H_2_O (Alpha Aesar 99.9%), and sodium hydroxide (NaOH) were used.

*Material Preparation*: Nanocrystalline pure CeO_2_ and Ce_1−*x*_Gd_*x*_O_2_ (*x* = 0.02, 0.04, 0.06, 0.08, and 0.10) samples were synthesized using coprecipitation method. The appropriate stoichiometric amount of (NH_4_)_2_Ce(NO_3_)_6_ and Gd(NO_3_)_3_·6H_2_O were used for synthesizing Ce_1−*x*_Gd_*x*_O_2_ NPs. Initially, (NH_4_)_2_Ce(NO_3_)_6_ and Gd(NO_3_)_3_·6H_2_O precursor solution was prepared in distilled water with magnetic stirring at the rate of 600 rpm. Then NaOH solution was added drop by drop to this solution until the pH level reached about 11. This solution was stirred about 4 h and then the synthesized pale‐yellow precipitate was collected. The precipitate was dried at room temperature and annealed in the furnace about 500 °C for 8 h. A set of samples, i.e., pure CeO_2_ and Ce_1−*x*_Gd_*x*_O_2_ (*x* = 0.02, 0.04, 0.06, 0.08, and 0.10) were prepared. The main chemical reactions during the experimental process are as follows

(27) $$6{\mathrm{NaOH}}_{\left(S\right)}\to 6{\mathrm{Na}}_{\left(\mathrm{aq}\right)}^{+}+\;6{\mathrm{OH}}_{\left(\mathrm{aq}\right)}^{-}$$

(28) $${\left({\mathrm{NH}}_{4}\right)}_{2}\mathrm{Ce}{\left({\mathrm{NO}}_{3}\right)}_{6}\to {\mathrm{Ce}}_{\left(\mathrm{aq}\right)}^{\mathrm{3+}}\;+\;{\mathrm{6NO}}_{3\;\left(\mathrm{aq}\right)}^{-}\;+\;{\mathrm{2NH}}_{4\;\left(\mathrm{aq}\right)}^{+}$$

(29) $$6{\mathrm{Na}}_{\left(\mathrm{aq}\right)}^{+}+\;6{\mathrm{NO}}_{3\;\left(\mathrm{aq}\right)}^{-}\to 6{\mathrm{NaNO}}_{3\;\left(\mathrm{aq}\right)}$$

(30) $$2{\mathrm{NH}}_{4\;\left(\mathrm{aq}\right)}^{+}+\;2{\mathrm{OH}}_{\left(\mathrm{aq}\right)}^{-}\to 2{\mathrm{NH}}_{3\;\left(g\right)}↑+\;2H_{2}O_{\left(\mathrm{aq}\right)}$$

(31) $${\mathrm{Ce}}_{\left(\mathrm{aq}\right)}^{3+}+\;4{\mathrm{OH}}_{\left(\mathrm{aq}\right)}^{-}+\;xH_{2}O_{\left(\mathrm{aq}\right)}\;\to \mathrm{Ce}{\left(\mathrm{OH}\right)}_{4}\cdot \;xH_{2}O_{\left(s\right)}↓$$

(32) $$\mathrm{Ce}{\left(\mathrm{OH}\right)}_{4}\cdot \;xH_{2}O_{\left(S\right)}\overset{\mathrm{at}\;\mathrm{RT}}{\to }\;\mathrm{Ce}{\left(\mathrm{OH}\right)}_{4\left(s\right)}+\;xH_{2}O_{\left(g\right)}↑$$

(33) $$\mathrm{Ce}{\left(\mathrm{OH}\right)}_{4\left(s\right)}\;\overset{500{\;}^{{}^{\circ}}C}{\to }\;{\mathrm{CeO}}_{2\;\left(s\right)}+\;2H_{2}O_{\left(g\right)}$$

The complete chemical reaction can be combined as

(34) $${\left({\mathrm{NH}}_{4}\right)}_{2}\mathrm{Ce}{\left({\mathrm{NO}}_{3}\right)}_{6}+\;6\mathrm{NaOH}\;\to \;\mathrm{Ce}{\left(\mathrm{OH}\right)}_{4}+\;6{\mathrm{NaNO}}_{3\;}+\;2{\mathrm{NH}}_{3\;}+\;2H_{2}O$$

(35) $$\mathrm{Ce}{\left(\mathrm{OH}\right)}_{4}\;\to {\mathrm{CeO}}_{2\;}+\;2H_{2}O$$

The final chemical reaction for the growth of various concentrations 2%, 4%, 6%, 8% and 10% of dopant Gd‐ions in CeO_2_ lattice are as follows

(36) $$\begin{array}{l} \left(1-x)\left[{\left({\mathrm{NH}}_{4}\right)}_{2}\mathrm{Ce}{\left({\mathrm{NO}}_{3}\right)}_{6}\right]+x[\mathrm{Gd}{\left({\mathrm{NO}}_{3}\right)}_{3}\cdot 6H_{2}O\right]\\ \;\;\;\;+\;6\mathrm{NaOH}\to {\mathrm{Ce}}_{1-x}{\mathrm{Gd}}_{x}O\;+\;\mathrm{molecular}\;\mathrm{gases}↑ \end{array}$$

*Nanomaterial's Characterization*: The structural properties of all the samples were characterized using XRD measurements on a Brucker D8 Advance diffractometer with Cu Kα radiation (λ = 1.5406 Å). The diffraction patterns were recorded at room temperature in the 2θ range from 10° to 90°. The surface morphology, particle size, and crystallinity of the samples were studied using TEM with Technai G2 20 S‐TWIN (FEI Netherlands) instrument operating at an accelerating voltage of 200 kV. Samples for the TEM investigation were prepared by dispersing the nanopowder in ethanol using an ultrasonicator to produce a dilute suspension. Then a standard holey carbon film supported on Cu grid was immersed in the suspension to produce the TEM sample. The particle size distribution was calculated for a total 150 number of particles using imagej software for TEM images. The optical characterizations were carried out by using SERS. For collecting Raman spectra, SERS of make Thermo Scientific DXRxi Raman Imaging Microscope with charge injection device detector using green laser with 532 nm excitation light source with its power kept at 10 mW were used. The UV–vis–NIR absorbance spectra on the samples in the wavelength range of 200–1000 nm with BaSO_4_ as standard were recorded employing a Shimadzu UV‐3600 Plus spectrophotometer with an integrating sphere. XPS spectra were recorded on a ultrahigh vacuum based Omicron Multiprobe Surface analysis System (Germany, Gmbh) operating at a base pressure of 5 × 10^−11 ^Torr. Mg Kα radiation source (with energy of 1253.6 eV) was used for data acquisition. An OMICRON EA125 hemispherical analyzer equipped with a 7 channeltron parallel detection unit was used to collect the XPS spectra. The calibration of binding energy in photoemission spectra was done referring to standard Au 4f_7/2_ emission line with energy resolution of ≈0.9 eV FWHM on Au 4f_7/2_ with pass energy of 20 eV during the measurement. The XPS core level data were analyzed after necessary carbon corrections for the Fermi energy referencing. The magnetic properties of the samples were investigated at room temperature using a Quantum Design MPMS‐3 SQUID system. The magnetization measurements were conducted by varying the applied field from −1.5 T to +1.5 T.

Photocatalytic cleavage of the water for hydrogen generation was carried out using the powder of photocatalytic molecular device (0.3 g powder of Pt/CeO_2_ or Pt/Gd*_x_*Ce_1−_ *_x_*O_2_ or CeO_2_ or Gd*_x_*Ce_1−_ *_x_*O_2_) that was suspended in 120 mL of aqueous hole‐scavenger electrolyte (20% CH_3_OH; pH = 7.0) in a reaction cell, under the irradiation of 1 sun (100 mW cm^−2^, AM1.5 G) visible light. The powder of the photocatalyst (0.2 g with and without Pt loading) was suspended in 120 mL of aqueous electrolyte (20% CH_3_OH pH = 7.0) in a double walled‐Pyrex glass reaction cell (volume ≈150 mL, with water jacket) that was sealed with a rubber septum and plastic wire lock.^111^, ^112^ Prior to start the photochemical reaction, the suspension was continuously purged with Ar for 1 h by maintaining the 1 atm pressure of the inner jacket solution for expelling the air content from the solution. Circulating water bath is used to maintain the temperature of the outer jacket at 25 °C. Afterward, the suspension was irradiated with a 300 W Xe lamp (>420 nm, light intensity 1 × 10^22^ photons per hour Xe lamp‐HX1, Model PE300UV, ISS). All the experiments were carried out under ambient conditions. Photocatalytic responses were hourly monitored in terms of the amount of hydrogen generated at 1–4 h time intervals. Hydrogen gas has very small density and not soluble in water. Therefore, the evolved hydrogen was collected into the inverted gas collection graduated bottle by displacement of water from a container. The collected gas was checked with the gas chromatograph (Shimazdu, Japan, thermal conductivity detector and molecular sieve with 5 A columns) throughout the course of the reaction.

## Conflict of Interest

The authors declare no conflict of interest.
